# Identification of BgP, a Cutinase-Like Polyesterase From a Deep-Sea Sponge-Derived Actinobacterium

**DOI:** 10.3389/fmicb.2022.888343

**Published:** 2022-04-12

**Authors:** Clodagh M. Carr, Bruno Francesco Rodrigues de Oliveira, Stephen A. Jackson, Marinella Silva Laport, David J. Clarke, Alan D. W. Dobson

**Affiliations:** ^1^School of Microbiology, University College Cork, Cork, Ireland; ^2^SSPC-SFI Research Centre for Pharmaceuticals, University College Cork, Cork, Ireland; ^3^Instituto de Microbiologia Paulo de Góes, Universidade Federal do Rio de Janeiro, Rio de Janeiro, Brazil; ^4^Departamento de Microbiologia e Parasitologia, Instituto Biomédico, Universidade Federal Fluminense, Niterói, Brazil; ^5^Environmental Research Institute, University College Cork, Cork, Ireland

**Keywords:** polyesterase, cutinase, PETase, plastic, marine

## Abstract

Many marine bacteria produce extracellular enzymes that degrade complex molecules to facilitate their growth in environmental conditions that are often harsh and low in nutrients. Marine bacteria, including those inhabiting sea sponges, have previously been reported to be a promising source of polyesterase enzymes, which have received recent attention due to their potential ability to degrade polyethylene terephthalate (PET) plastic. During the screening of 51 marine bacterial isolates for hydrolytic activities targeting ester and polyester substrates, a *Brachybacterium ginsengisoli* B129SM11 isolate from the deep-sea sponge *Pheronema* sp. was identified as a polyesterase producer. Sequence analysis of genomic DNA from strain B129SM11, coupled with a genome “mining” strategy, allowed the identification of potential polyesterases, using a custom database of enzymes that had previously been reported to hydrolyze PET or other synthetic polyesters. This resulted in the identification of a putative PET hydrolase gene, encoding a polyesterase-type enzyme which we named BgP that shared high overall similarity with three well-characterized PET hydrolases—LCC, TfCut2, and Cut190, all of which are key enzymes currently under investigation for the biological recycling of PET. *In silico* protein analyses and homology protein modeling offered structural and functional insights into BgP, and a detailed comparison with Cut190 revealed highly conserved features with implications for both catalysis and substrate binding. Polyesterase activity was confirmed using an agar-based polycaprolactone (PCL) clearing assay, following heterologous expression of BgP in *Escherichia coli*. This is the first report of a polyesterase being identified from a deep-sea sponge bacterium such as *Brachybacterium ginsengisoli* and provides further insights into marine-derived polyesterases, an important family of enzymes for PET plastic hydrolysis. Microorganisms living in association with sponges are likely to have increased exposure to plastics and microplastics given the wide-scale contamination of marine ecosystems with these plastics, and thus they may represent a worthwhile source of enzymes for use in new plastic waste management systems. This study adds to the growing knowledge of microbial polyesterases and endorses further exploration of marine host-associated microorganisms as a potentially valuable source of this family of enzymes for PET plastic hydrolysis.

## Introduction

Marine microbial communities play a central role in maintaining and supporting ocean ecosystems, largely by participating in biogeochemical processes, such as carbon cycling (Arnosti et al., 2014). Moreover, by harnessing their ability to catalyze biological reactions, marine microorganisms can be used to reduce environmental pollutants, as well as the damaging effects of industrial activities (Carr et al., 2020; de Oliveira et al., 2020). Many marine enzymes offer novel biocatalytic properties, particularly when compared to those from terrestrial environments, due to the diversity of conditions in which they normally operate and the presence of unusual and often bulky substrates with distinct chemical substituents in marine ecosystems (Trincone, 2011, 2017; Parages et al., 2016).

The plastics era, which evolved alongside fast-paced, modern lifestyles and transformative technological advancements, has contributed to a major shift toward a society of mass production and excess consumption. Over the past decade, 4.8–12.7 million metric tons (MMT) of plastic has been reported to enter the world’s oceans on an annual basis, a figure which could accumulate to 250 MMT by 2025 (Yakimov et al., 2022). The “plastisphere,” which refers to the microbial communities colonizing plastic debris, has attracted particular attention in the context of marine ecosystems (Amaral-Zettler et al., 2020; Li et al., 2021; Yakimov et al., 2022), with recent multi-omics analyses on the plastisphere highlighting this habitat as a promising source of plastic-degrading microorganisms (Suzuki et al., 2021; Wright et al., 2021; Zrimec et al., 2021).

Plastics are as versatile and easy to produce as they are problematic and difficult to discard. Unlike the broadly employed mechanical recycling methods that result in plastic “downcycling,” chemical and biological recycling processes aim to facilitate virgin plastic production by recovering the original monomers (Carr et al., 2020). Thus, biological-based methods in plastic disposal or recycling strategies are gaining increased attention (Bahl et al., 2021). These methods, which may be either enzyme- or whole-cell catalyzed, are particularly attractive as they offer the possibility of comparatively mild reaction conditions, high specificity, and reduced energy requirements and greenhouse gas emissions, that are potentially more cost-effective when compared to chemical recycling (Carr et al., 2020; Singh et al., 2021).

Polyethylene terephthalate (PET) is a crude-oil derived thermoplastic synthetic polyester, consisting of terephthalic acid (TPA) and ethylene glycol (EG) monomers (Webb et al., 2013). PET, which is currently the most abundantly used polyester plastic in the world, has a predicted life span of up to 50 years (Pirillo et al., 2021). Its widespread use across the packaging, textile, automotive, electrical, and electronics sectors is primarily related to its light weight and high mechanical strength, together with its insulating properties, and capacity to act as both a gas and moisture barrier (Webb et al., 2013; Liu et al., 2019a; Dissanayake and Jayakody, 2021). In particular, its durability and moldability are convenient for the production of PET containers, films, and fibers, together with bottles commonly used for carbonated soft drinks and water. However, the aforementioned qualities that make PET an attractive material for various industrial applications are also responsible for the problems it can cause if its disposal is mismanaged (Jaiswal et al., 2020). The backbone of the PET polymer is highly stable, which together with its surface hydrophobicity and crystallinity, restricts its natural breakdown (Liu et al., 2019a; Kawai et al., 2020).

Microbial enzymes with PET depolymerizing activity have however introduced the possibility of PET degradation or modification to allow the development of techno-economically feasible, microbial-based PET recycling processes (Tournier et al., 2020; Zimmermann, 2020). These PET-active enzymes commonly belong to carboxylesterase, lipase, and cutinase families (Danso et al., 2019), and to date have predominantly been identified in thermophilic Actinobacteria, particularly in the genus *Thermobifida* (Silva et al., 2011; Wei and Zimmermann, 2017). The best studied enzyme in this area is a PET hydrolase or “PETase” from *Ideonella sakaiensis* 201-F6 (*Is*PETase), on the basis that the strain was isolated from a PET-enriched environment and had potentially evolved specifically to process PET. This enzyme was subsequently biochemically and structurally characterized and adapted across a number of studies, in an effort to enhance its overall activity and stability (Han et al., 2017; Chen et al., 2018; Joo et al., 2018; Liu et al., 2019a; Urbanek et al., 2020).

Given the aforementioned studies highlighting the plastisphere as a promising source of plastic-degrading microorganisms, coupled with the fact that plastic constitutes up to 90% of the solid waste found in oceans (Oliveira et al., 2020); it is perhaps not surprising that microorganisms from marine ecosystems and holobionts, such as marine sponges and seaweeds, are likely to have been exposed to plastics or microplastics in marine environments and can be targeted as a potential source of PET-active enzymes. Our group has recently reported on a PETase-like enzyme with polycaprolactone (PCL)-degrading activity, which was identified in the marine sponge-derived *Streptomyces* sp. SM14 strain (Almeida et al., 2019). Additionally, a putative polyesterase named MorEst, from an Antarctic psychrotrophic bacterium from the genus *Moraxella* sp. has been reported to degrade a range of polyesters, including PCL (Nikolaivits et al., 2020). Also, a novel polyester hydrolase from the marine bacterium *Pseudomonas aestusnigri* with activity against PET film has been reported which, following mutagenesis, also displayed some activity against commercial PET bottles (Bollinger et al., 2020). Furthermore, a study of marine metagenomic datasets has reported that genes encoding PET hydrolases are globally distributed in marine environments (Danso et al., 2018; Zrimec et al., 2021). Thus, marine microbiomes appear to be a potentially rewarding source of novel polyesterase and PETase enzymes and given the continued need to increase the diversity of enzymes and microorganisms acting on artificial polyesters, may prove a useful resource for these types of enzymes.

The deep sea, while one of the most vast biomes on our planet, still remains relatively unexplored. This environment, defined by seawater depths below 1,000 m and averaging at 3,800 m, is considered extreme; given its challenging physical conditions and unique composition (Jin et al., 2019). The microbial communities that can survive and thrive in the deep sea face a dynamic range of pressure, temperature, pH, and salinity, together with exposure to various chemicals and metals. The production of enzymes that tolerate these parameters contribute to the adaptive strategy of these microorganisms, and so the deep sea represents a promising source of stable and robust enzymes for industrial use (Jin et al., 2019). Furthermore, marine microbial communities and their hydrolytic enzymes may interact with synthetic polymer debris, within these ecosystems (Galloway et al., 2017). Plastic particles have been detected in deep-sea ecosystems, such as in Western Pacific Ocean sediment at depths between 4,601 and 5,732 m, where the most abundant microplastics were found to be poly(propylene-ethylene) copolymer (PP-PE) and PET (Zhang et al., 2020). It is worth noting that, although the ability to hydrolyze PET could potentially enhance the evolutionary fitness of microorganisms in the environment, this would require that PET is fully metabolized. In the case of the aforementioned *Ideonella sakaiensis*, the role of biofilms and microbial consortia, together with a dedicated PET metabolic pathway, have been explored to provide an explanation for its ability to grow on PET as a sole carbon source (Taniguchi et al., 2019; Carr et al., 2020).

Following the screening of a number of marine sponge-derived bacterial isolates, with lipolytic activity that was initially observed on tributyrin agar, and with polyesterase activity subsequently being confirmed on polycaprolactone diol (PCD) and polycaprolactone (PCL) agar plates; a *Brachybacterium ginsengisoli* B129SM11 strain isolated from the deep-sea sponge *Pheronema* sp. which had been sampled at a depth of 2,129 m, was prioritized for further analyses. Genome mining of the B129SM11 strain revealed a putative PETase gene which, following computational protein analyses and homology modeling, was shown to encode a cutinase-like enzyme, named BgP. Polyesterase activity was subsequently confirmed on ester and polyester substrates, following cloning and heterologous expression of BgP in *Escherichia coli*.

Cutinases and cutinase-like enzymes have become the focus of a number of polyester and PET hydrolysis studies, and are considered versatile biocatalysts with several uses, which are not limited to waste treatment and biorecycling, but also have other biotechnological applications, for example, in sustainable chemical synthesis, polymerization, and polymer modification (Chen et al., 2013; Nikolaivits et al., 2018; Molitor et al., 2020). This is the first report of a cutinase-like polyesterase being identified in a deep-sea sponge-derived *Brachybacterium* spp. isolate and this work provides further insight into enzymes for polyester degradation.

## Materials and Methods

### Sponge Sampling and Isolation of Bacterial Strains

The *R.V. Celtic Explorer* Irish research vessel and *Holland I* remotely operated vehicle (ROV) were employed for the collection of the marine sponge *Pheronema* sp., from the North Atlantic Ocean in the Irish Rockall Trough. The sponge sample was obtained from a depth of 2,129 m as part of a biodiscovery cruise in 2010. Following collection, sponge samples were rinsed with sterile artificial seawater (ASW) [Instant Ocean*™*, 3.33% (w/v)], macerated with a sterile razor blade, then placed in a tube with sterile glass beads and vortexed for 2 min. Sterile ASW was added, and samples were vortexed again. Dilution series were performed to 10^–5^ with sterile ASW and microbial cultures were isolated by spread plating 100 μL of each dilution onto the following growth media; (i) starch-yeast-peptone seawater agar (SYP-SW): 1% (w/v) starch, 0.4% (w/v) yeast extract, 0.2% (w/v) peptone, 3.33% (w/v) artificial sea salts (Instant Ocean*™*), 1.5% (w/v) agar; (ii) modified marine agar (MMA): 0.005% (w/v) yeast extract, 0.05% (w/v) tryptone, 0.01% (w/v) β-glycerol phosphate disodium salt, pentahydrate, 3.33% (w/v) artificial sea salts (Instant Ocean*™*), 1.5% (w/v) agar, and (iii) chitin agar: 4% (v/v) colloidal chitin, 1.5% (w/v) agar.

### Enzyme Activity Screening

As part of a wider polyesterase screening study investigating 51 strains from a variety of marine sources, isolate B129SM11 from *Pheronema* sp. sponge was tested for enzyme activities against the following substrates: tributyrin (glyceryl tributyrate, Sigma Aldrich), polycaprolactone diol (PCD, Sigma Aldrich), and polycaprolactone (PCL, Sigma Aldrich), using agar-based clearing assays previously detailed in Molitor et al. (2020), with the following adaptations; marine agar (3.74% Marine Broth 2216, BD Difco*™*; 1.5% agar, Sigma) was supplemented with tributyrin (1.0%), PCD (3.0%), or PCL (0.1%). A Waring^®^ laboratory blender was used to emulsify the tributyrin and PCD substrates with the media before autoclaving. PCL pellets were dissolved in acetone at 70°C before adding dropwise, under fast stirring to autoclaved agar. Cultures were spot inoculated onto screening plates and incubated at 28°C, then checked daily for zones of clearing for up to 7 days. Based on the observed activities, confirmed 16S rRNA gene identities, and considering its deep-sea origin, *Brachybacterium ginsengisoli* B129SM11 was subsequently selected as a suitable candidate for genome sequencing (Table 1).

**TABLE 1:** Confirmed hydrolytic activities for five isolates derived from deep-sea sponges, which were screened on ester (tributyrin) and polyester (PCD and PCL) substrates to identify potential polyesterase producers.

Isolate ID	Sponge host	16S rRNA gene	Depth (m)	Tributyrin	PCD	PCL
B129SM11	*Pheronema* sp.	*Brachybacterium ginsengisoli*	2,129	+	+	+
B226SK6	*Inflatella pellicula*	*Micrococcus* sp.	2,900	+	+	+
B226M5	*Inflatella pellicula*	*Agreia* sp.	2,900	+	+	−
B98C26	*Hexactinellida* sp.	*Jiella aquimaris*	1,480	+	+	−
B98SN116	*Hexactinellida* sp.	*Tsukamurella pseudospumae*	1,480	+	−	−

*Sponge sample collection depths and sponge host taxonomic identities are provided, along with 16S rRNA gene molecular identification for each bacterial isolate.*

### Genomic DNA Extraction

Genomic DNA (gDNA) was extracted from 5 mL cultures grown in Marine Broth 2216 for 24 h at 30°C, with shaking (125 rpm). Cells were pelleted by centrifugation (Eppendorf Centrifuge 5804R) at 4400 x *g* for 20 min, then broth supernatants were discarded, and cell pellets were allowed to drain. The method used to obtain gDNA from isolate B129SM11 was based on a previously described phenol-chloroform-isoamyl alcohol extraction procedure (Jackson et al., 2018).

### Genome Sequencing, Assembly, and Annotation

Next-generation sequencing was completed by Eurofins Genomics (Konstanz, Germany) using Illumina HiSeq technology (NovaSeq 6000 sequencing system), including library preparation and initial quality checks. FastQC (v 0.11.9)^1^ was used to evaluate the quality of the raw sequence reads. Reads were then assembled *de novo* using SPAdes Genome Assembler (v 3.15.0) (Bankevich et al., 2012), excluding contigs <400 bp. QUAST (v 5.0.2) (Gurevich et al., 2013) was employed to assess the overall quality of the final assembly, and completeness and contamination were determined using CheckM (v 1.1.3) (Parks et al., 2015). Initial annotation was completed using Prokka (v 1.14.6) (Seemann, 2014), while functional annotation of the predicted protein output was carried out with eggNOG mapper (v 2.0), against the eggNOG database (v 5.0) (Huerta-Cepas et al., 2019), and with the BLASTKOALA (KEGG Orthology And Links Annotation) tool using the KEGG (Kyoto Encyclopedia of Genes and Genomes) database (Kanehisa et al., 2016). The Genome Database Taxonomy Toolkit (GTDB-Tk, v 1.5.0) was applied for phylogenomic analyses, operating based on an established set of single-copy conserved marker genes (Chaumeil et al., 2020).

### Genome Mining

A reference dataset, containing the amino acid sequences of 26 PET hydrolases and homologous polyesterases, was used to produce a custom BLASTP database with the makeblastdb command-line tool (Table 2), following a previously described strategy for genome mining (Almeida et al., 2019). Potential homologs were identified in the genome of B129SM11 by performing a BLASTP search of the annotated Prokka output against the constructed database, employing an e-value threshold of 1e-30. The specific homology search results generated for BgP with three well-studied PET-hydrolyzing cutinases are presented in Table 3.

**TABLE 2:** Reference dataset of functionally verified polyesterases having activity on PET or PET model substrates, which was used to conduct BLASTP protein homology searches against the B129SM11 annotated genome and for further phylogenetic inferences.

Enzyme	Source	Uniprot accession	References
TfH	*Thermobifida fusca* DSM43793	Q6A0I4	Müller et al., 2005
Tfu_0882	*Thermobifida fusca* YX	Q47RJ7	Chen et al., 2008
Tfu_0883	*Thermobifida fusca* YX	Q47RJ6	Chen et al., 2008
TfCut1	*Thermobifida fusca* KW3	E5BBQ2	Herrero Acero et al., 2011
TfCut2	*Thermobifida fusca* KW3	E5BBQ3	Herrero Acero et al., 2011
Est1	*Thermobifida alba* AHK119	D4Q9N1	Thumarat et al., 2015
Est119	*Thermobifida alba* AHK119	F7IX06	Hu et al., 2010
Thc_Cut1	*Thermobifida cellulosilytica* DSM44535	E9LVH8	Herrero Acero et al., 2011
Thc_Cut2	T*hermobifida cellulosilytica* DSM44535	E9LVH9	Herrero Acero et al., 2011
Thf42_Cut1	*Thermobifida fusca* DSM44342	E9LVI0	Herrero Acero et al., 2011
Tha_Cut1	*Thermobifida alba* DSM43185	E9LVH7	Ribitsch et al., 2012a
Thh_Est	*Thermobifida halotolerans* DSM44931	H6WX58	Ribitsch et al., 2012b
LCC	Metagenome from leaf-branch compost	G9BY57	Sulaiman et al., 2012
Tcur1278	*Thermonospora curvata* DSM43183	D1A9G5	Wei et al., 2014
Tcur0390	*Thermonospora curvata* DSM43183	D1A2H1	Wei et al., 2014
Cut190	*Saccharomonospora viridis* AHK190	W0TJ64	Kawai et al., 2014
*Is*PETase	*Ideonella sakaiensis* strain 201-F6	A0A0K8P6T7	Yoshida et al., 2016
BhrPETase	Thermophilic bacterium strain HR29	A0A2H5Z9R5	Xi et al., 2021
SM14est	*Streptomyces* sp. SM14 (marine)	DAC80635.1 (Genbank)	Almeida et al., 2019
PE-H	*Pseudomonas aestusnigri* (marine)	A0A1H6AD45	Bollinger et al., 2020
BsEstB	*Bacillus subtilis* 4P3-11	D7R6G8	Ribitsch et al., 2011
PET12	*Polyangium brachysporum*	A0A0G3BI90	Danso et al., 2018
PET2	Uncultured bacterium (marine metagenome)	C3RYL0	Danso et al., 2018
PET5	*Oleispira antarctica* RB-8	R4YKL9	Danso et al., 2018
PET6	*Vibrio gazogenes*	A0A1Z2SIQ1	Danso et al., 2018
HiC	*Humicola insolens*	A0A075B5G4	Ronkvist et al., 2009

**TABLE 3:** Sequence similarities between BgP (290 aa) and bacterial polyesterases of interest for PET biorecycling, generated by a BLASTP search of annotated *B. ginsengisoli* B129SM11 proteins.

Protein	Microbial source	Score (bits)	*E*-value	Identities (%)	Length (aa)
Cut190	*Saccharomonospora viridis* AHK190	312	9e-111	62	304
TfCut2	*Thermobifida fusca* KW3	288	9e-102	59	261
LCC	Leaf-branch compost metagenome	270	3e-94	56	293

*aa, amino acids.*

### Protein Analysis and Homology Modeling

Phylogenetic analysis of protein sequences was carried out using the MEGA-X program (Kumar et al., 2018) with the maximum likelihood statistical method and the WAG + G model, under 100 bootstrap replications and a 50% bootstrap cut-off value. T-COFFEE Expresso (Di Tommaso et al., 2011) was employed to generate amino acid sequence alignments, and outputs were graphically represented and analyzed using ESPript 3.0 (Gouet et al., 1999). The Lipase Engineering Database (LED) BLAST resource facilitated the identification of the catalytic triad and oxyanion hole residues.^2^ The native protein signal peptide and corresponding cleavage site were predicted using the SignalP 5.0 server (Armenteros et al., 2019). Subcellular localization was inferred with PSORTb (Yu et al., 2010), Gpos-mPLoc (Shen and Chou, 2009), and FUEL-mLoc (Wan et al., 2017). The ExPASy-ProtParam tool was applied to predict physico-chemical properties such as molecular weight, theoretical isoelectric point (pI), amino acid composition, together with aliphatic and instability indexes (Gasteiger et al., 2005). InterProScan,^3^ Pfam,^4^ and SUPERFAMILY^5^ databases were applied for protein family classification and to uncover functional protein domains.

The SWISS-MODEL homology-modeling server was run to predict a three-dimensional (3D) structure for the BgP protein (Waterhouse et al., 2018). A cutinase-like lipase, SeL, from *Streptomyces exfoliatus* (PDB Accession Code: 1JFR) (Wei et al., 1998), served as a template for model construction. Model quality was initially assessed from SWISS-MODEL parameters, followed by validation using ERRAT (Colovos and Yeates, 1993), VERIFY3D (Eisenberg et al., 1997), and PROCHECK (Laskowski et al., 2006), which are tools within the Structure Analysis and Verification Server 6.0 (SAVES^6^), in addition to the ProSA-web server (Wiederstein and Sippl, 2007). The active site pocket and potential binding residues were detected using Phyre2 (Colovos and Yeates, 1993) together with 3DLigandSite (Wass et al., 2010). Protein stability was predicted by SCooP (v 1.0), using a temperature-dependent dataset containing mesostable and thermostable proteins (Pucci et al., 2017). UCSF-Chimera was used to visualize the 3D model and enable structural comparison with the known polyesterase Cut190 (PDB code: 4WFI) (Pettersen et al., 2004).

### Cloning and Heterologous Expression

Snapgene (v 5.2.5.1) software^7^ was employed to assist primer design and to simulate cloning. The GenScript Restriction Enzyme Map Analysis Tool^8^ and the Molbiotools Restriction Analyzer^9^ were used to assess the native gene sequence and aid in the selection of restriction enzymes. The Sigma-Aldrich OligoEvaluator*™*^10^ and the Thermo Fisher Scientific Multiple Primer Analyzer^11^ were run to predict possible secondary structures or primer-dimer pairings and revealed the overall stability, melting temperature (T_m_), and GC content. The New England BioLabs (NEB) T_m_ Calculator^12^ was used to evaluate specific annealing and melting temperatures for use of these primers with Q5^®^ High-Fidelity Polymerase (NEB). Primer binding and PCR product length were predicted by uploading the *B. ginsengisoli* B129SM11 genome to Primer-BLAST (Ye et al., 2012).

The *bgp* gene was amplified from genomic DNA with a forward primer (5′-AAAAACATATGCACGCACAGAC CCGCAGGATC-3′), containing an *Nde*I restriction site that replaces the gene sequence start codon, and a reverse primer (5′-AAAAAGCGGCCGCTTAATGGTGGTGGTGATGGTGGAAC GGGCAGGTGGACCGGAC-3′), which incorporates a C-terminal His-Tag, a stop codon, and a *Not*I restriction site. The predicted BgP signal peptide was maintained in the construct design. The PCR product was cloned into the pET20b(+) plasmid (Novagen^®^), resulting in the pET20b(+):BgP vector construct, which was transformed into NEB^®^ 5-alpha competent *E. coli* (New England Biolabs) for storage. The vector was subsequently conjugated into *E. coli* BL21-Codon Plus (DE3)-RIPL (Agilent Technologies) for heterologous protein expression. The insert was confirmed by a diagnostic restriction digest of Miniprep (Qiagen) purified plasmid, and by colony PCR, followed by Sanger sequencing of the amplified product (Eurofins LightRun).

Single colonies of the BL21 RIPL-(pET20b:BgP) recombinant clone were inoculated into 1 mL aliquots of Luria Bertani broth supplemented with 0.003% (w/v) chloramphenicol and 0.01% (w/v) ampicillin, and incubated at 37°C overnight with shaking (225 rpm). After 14 h, 50 μL was subcultured in fresh 1 mL aliquots of LB (no antibiotic selection) and incubated for 2 h again at 37°C with shaking. Activity was confirmed on LB agar plates containing 1% tributyrin and PCD, and 0.1% PCL, following inoculation of 10 μL spots onto the plates, which were then incubated for 6 days and monitored carefully for substrate clearance.

## Results and Discussion

### Screening for Polyesterase Activities in Marine-Derived Bacterial Isolates

A recent report on the plastisphere of deep-sea samples in the Southwest Atlantic Ocean, involving the long-term colonization of plastic substrates by deep-sea microbes, has identified taxa in the core microbiome that may be related to plastic degradation. It also highlighted the fact that viable strains can be recovered from deep-sea conditions, which have the potential to be exploited for their plastic-degradation capacity (Agostini et al., 2021). A further indication of the potential of the marine environment as a source of novel polyesterase genes is reflected in a study of marine metagenomic datasets which reported that genes encoding PET hydrolases are globally distributed in marine environments (Danso et al., 2018). The capacity of marine microorganisms to degrade a range of polyesters is likely to be as a result of the high levels of exposure to plastics and microplastics that they have been and continue to be exposed to; with estimates indicating that over 250 thousand tons of plastic are currently floating in the oceans (Eriksen et al., 2014).

Previous reports have also highlighted the ability of marine-derived bacteria to degrade synthetic plastics, including *Bacillus* and *Rhodococcus* strains, together with a MorEst polyesterase from an Antarctic psychrotrophic bacterium which was able to degrade a range of polyesters, as well as a *Pseudomonas aestusnigri* isolate with a novel polyester hydrolase activity against PET film (Auta et al., 2018; Bollinger et al., 2020; Nikolaivits et al., 2020). Screening of marine metagenomic data revealed an esterase, GEN0105, that was shown to hydrolyze bis(benzoyloxyethyl)-terephthalate (i.e., 3PET), along with polylactic acid (PLA), and PCL (Hajighasemi et al., 2018). Most recently, a bacterial consortium containing three marine bacterial species, namely *Exiguobacterium* sp., *Halomonas* sp., and *Ochrobactrum* sp. has been reported to degrade PET film with recombinant hydrolases and esterase genes from these strains demonstrating strong PET film degradation effects when heterologously expressed in *E. coli* (Gao and Sun, 2021). Thus, bacteria from marine environments represent a good source of polyesterases, which we should begin to sustainably exploit in the future.

Marine sponges (phylum Porifera) are generally sessile marine filter feeders, with some motile and carnivorous exceptions (Wilkinson, 1978; Lavrov and Kosevich, 2018; Vacelet, 2020). They harbor an abundant and diverse range of microbial symbionts, which can be responsible for up to 35% of the sponge biomass (Vacelet, 1975). Given that marine sponges, such as the deep-sea sponge *Pheronema* sp., filter large quantities of seawater (up to 24,000 L of water per day/Kg sponge) to obtain nutrients (Vacelet, 1975; Taylor et al., 2007), coupled with the fact that with typical densities of bacteria in seawater of up to 10^6^ cells/mL, then there is the potential for sponges to ingest a total of 2.4 × 10^13^ bacterial cells on a daily basis (Hill, 2003). Thus, deep-sea sponges are likely to be a good source of such marine bacteria, with a number of bacteria from different genera having been isolated from them (Romanenko et al., 2008; Xin et al., 2011; Borchert et al., 2017; Williams et al., 2020).

There is clear evidence that deep-sea environments are exposed to microplastics, as evidenced by a report of the presence of a microplastic fiber very similar to PET in the shrimp *Eurythenes plasticus* recovered from depths of between 6010 and 6949 in the Mariana Trench in the Northwest Pacific Ocean (Weston et al., 2020). Thus, given the high levels of plastics and microplastics in the oceans, marine sponges are likely to be a good source of bacteria with potential to degrade polyesters. This is borne out by the fact that we recently reported on a PETase-like enzyme with polycaprolactone (PCL)-degrading activity which was identified in *Streptomyces* SM14 strain which was isolated from the sponge *Haliclona simulans* (Almeida et al., 2019).

A total of 51 bacterial strains that had previously been isolated from various marine sources, including shallow sea lough sponges (Jackson et al., 2012; Margassery et al., 2012), deep-sea sponges, and seaweeds (unpublished), were screened for polyesterase activities using tributyrin and PCD substrates, with either promising or interesting isolates subjected to further screening on PCL (Molitor et al., 2020). Five deep-sea sponge isolates had their activities confirmed on the three substrates, with *Brachybacterium ginsengisoli* strain B129SM11 and *Micrococcus* sp. strain B226SK6 showing the best range of activities (Table 1). The isolate B226SK6 from *Inflatella pellicula* targeted tributyrin more specifically, with fainter activity toward the polyester substrates, whereas the isolate B129SM11 from *Pheronema* sp. sponge displayed superior activity across all three substrates. Based on these activities, the B129SM11 strain was chosen for genome sequencing and subsequent genome mining, in an effort to identify genes potentially involved in PCL degradation.

Tributyrin is a short-chain triglyceride that served as a suitable substrate for preliminary screening, given that polyesterases also display lipolytic activity. For more specific targeting of polyesterases, the aliphatic polyesters PCD and PCL were employed. Although these are aliphatic polyesters, with a simple composition compared to aliphatic-aromatic copolyesters like PET, many polyesterase enzymes have been reported to have activity toward both substrate types (Molitor et al., 2020).

The genus *Brachybacterium* is a high GC member of the *Dermabacteraceae* family, within the phylum Actinobacteria. *Brachybacterium* species have previously been identified from various environments including oil-contaminated coastal sand, lake sediment, and more recently from deep-sea sediments in the Southern Ocean (Zhao et al., 2017; Ziganshina et al., 2018). *Brachybacterium* species have previously been reported in association with sponges (Kiran et al., 2014; Liu et al., 2019b), including deep-sea sponges from the Antarctic (Xin et al., 2011). While marine Actinobacteria have been reported to be capable of remediation of environmental pollutants including petroleum hydrocarbons and plastics (Rathore et al., 2021); members of the genus *Brachybacterium* have also been shown to possess the ability to degrade alkanes, phenols, and naphthalene (Velmurugan and Arunachalam, 2009; Wang et al., 2010, 2014). In addition, following the screening of Actinobacteria from a marine lake, a *Brachybacterium* sp. isolate was identified which displayed high lipolytic activity (Sadati et al., 2021). Thus, *Brachybacterium ginsengisoli* B129SM11 from the deep-sea sponge *Pheronema* sp. was expected to be an interesting candidate for further investigation in the context of polyester degradation.

### Genome Analyses and Mining of *Brachybacterium ginsengisoli* B129SM11

Genome mining, in combination with computational tools and dedicated databases, allows for the identification of new homologs of enzymes of biotechnological interest and offers an efficient means to discover potential novel biocatalysts (Zaparucha et al., 2018). Recent examples of biocatalysts that have been uncovered using a genome mining-based approach include a thermostable monoacylglycerol lipase from a deep-sea *Geobacillus* sp. (Tang et al., 2019) and a salt-tolerant, enantio-selective esterase from the actinobacterium *Dactylosporangium aurantiacum* that generated optically pure (R)-3-hydroxybutyrate (Wang et al., 2018).

Genomic DNA from *B. ginsengisoli* B129SM11 was sequenced using the Illumina HiSeq NGS system. The raw sequence data was evaluated in terms of overall quality prior to genome assembly and annotation. A high-quality draft genome was successfully assembled for strain B129SM11 in 22 contigs, with 100% completeness and very low contamination (0.58%). The assembled genome (3.98 Mb) displayed a high GC content of 71.55%. A total of 3,505 coding sequences (CDS) were annotated by Prokka (Supplementary Table 1). The B129SM11 genome properties are similar to those of other *Brachybacterium* spp. genomes deposited in the NCBI database in terms of genome size, GC content, and number of CDSs.

The genome of strain B129SM11 was mined for genes encoding potential PET polyesterases by first assembling a reference data set containing 26 PET hydrolyzing enzymes or homologs having functionally verified activities against some PET and/or synthetic polyester substrates (Table 2). This dataset was used to construct a database with the BLASTP suite tool to search against, including four thermophilic cutinases which have been recognized as the most promising candidates for PET biorecycling to date; HiC and LCC, together with TfCut2 and Cut190 mutants (Kawai et al., 2020) (Table 3). Cut190 and TfCut2 are actinobacterial enzymes, whereas LCC and HiC are derived from metagenomic and fungal sources, respectively.

BgP was identified by employing a BLASTP sequence similarity search of the Prokka-annotated protein output file generated for strain B129M11, against the polyesterase database. BgP produced significant alignments with 24 of the 26 enzymes, including Cut190, TfCut2, and LCC. BgP was annotated as a “poly(ethylene) terephthalate hydrolase,” although it cannot be assumed based on this preliminary description that BgP is a true PET hydrolase. The protein shared high amino acid identity (>55%) with LCC, TfCut2, and Cut190 (Table 3). Of these three enzymes, BgP shared the highest sequence identity, that is 62%, with Cut190 from *Saccharomonospora viridis* AHK190 (UniProt Accession: W0TJ64) (Kawai et al., 2014). Given that HiC is a fungal cutinase, it was not expected to produce as significant an alignment with BgP. The gene sequence for BgP was deposited in the GenBank database and can be found under the accession number ON000823.1 (Submission ID: 2562113).

### Computational Protein Analyses of BgP Enzyme

Synthetic polyester plastics may be hydrolyzed by microbial lipases, carboxylesterases, and cutinases, which can therefore be collectively referred to as polyesterases (Gricajeva et al., 2021). Many polyesterases are PET hydrolyzing enzymes (PHEs), although cutinases are considered most effective in cleaving polyester bond linkages (Kawai et al., 2020). It has been suggested that PHEs should be categorized either as PET-surface modifying enzymes or PET hydrolases, based on their differing abilities to hydrolyze the inner building blocks of PET. The PET hydrolase group can be further divided into mesophilic and thermophilic members, with thermophilic PETases deemed more suitable for application in the biological recycling of PET, given that this process requires temperatures between 65 and 75°C where the polymer is flexible enough for enzyme access. On the other hand, mesophilic enzymes resembling *Is*PETase from *I. sakaiensis* 201-F6, which have been found in both marine and terrestrial environments, are deemed more appropriate for the decomposition of PET waste. However, this presents its own challenges, for instance when environmental temperatures are not compatible with the enzyme employed (Kawai et al., 2019, 2020; Kawai, 2021). There are four thermophilic PET hydrolases that have been considered appropriate for PET biorecycling; metagenomic LCC, and variants of Cut190 and TfCut2 from actinomycetes (Table 3), along with fungal HiC. More recently, two thermophilic polyesterases from metagenomic sources, namely, PHL7 (Sonnendecker et al., 2021) and BhrPETase (Xi et al., 2021) have emerged as promising candidates, with highly efficient activities toward amorphous PET films.

BgP was classified as an α/β hydrolase member using InterProScan, Pfam, and SUPERFAMILY. Most polyesterases fall into this α/β hydrolase superfamily, which is comprised of enzymes that are structurally similar, yet functionally diverse (Gricajeva et al., 2021). Based on the results generated from Gpos-mPLoc and FUEL-mLoc, the enzyme appears to be extracellular. SignalP predicted a Sec/SPI-type signal peptide (first 30 AA) for standard secretion, with a likelihood of 0.9571. A potential cleavage site (AFA-AD) was identified between positions 31 and 32, based on the probability value of 0.9741.

**FIGURE 1:**
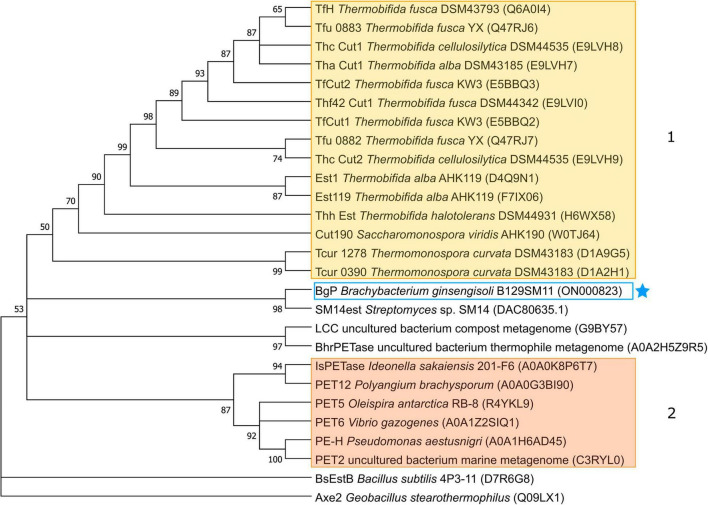
Maximum-likelihood (ML) phylogenetic tree of BgP among known PET-hydrolyzing enzymes and homologs from the reference dataset. Bootstrap consensus was inferred with 100 replicates, displaying only values above 50%, and the best-fit substitution model for this tree was WAG + G. Two main taxonomic clades are indicated; (1) proteins from thermophilic Actinobacteria (shaded in yellow) and (2) proteins from psychrophilic or mesophilic Proteobacteria (shaded in orange). BgP (starred) is tightly clustered with marine sponge-derived SM14est. LCC and BhrPETase from metagenomic thermophiles are also clustered together. A *Geobacillus stearothermophilus* acetylxylan esterase, Axe2 (UniProt Accession Number: Q09LX1) served as an outgroup.

In an effort to gain further insights into the potential functional and evolutionary relatedness of BgP to known polyesterases, a phylogenetic tree was constructed with protein sequences of BgP and PET-hydrolyzing enzymes or their homologs, using MEGA-X with CLUSTALW sequence alignment (Figure 1; Thompson et al., 1994). HiC is phylogenetically distinct due to its fungal origin and was therefore excluded from our phylogenetic reconstruction. All enzymes in the tree appear to originate from a common ancestor, with the exception of BsEstB, a PET hydrolyzing *p*-nitrobenzylesterase that is structurally different from most α/β hydrolases with a sequence length > 400 bp and where glutamate replaces the more commonly observed aspartate of the polyesterase catalytic triad. Proteins derived from thermophilic Actinobacteria are seen to form one clade, descending from the same node, while proteins from Proteobacteria comprise another clade, with these two clades labeled 1 and 2, respectively (Figure 1). Our polyesterase, BgP, appears to be located closer to clade 1, although derived from a different node, where it groups with SM14est from the *Haliclona simulans* sponge. Given that BgP and SM14est are also actinobacterial enzymes, their separation from clade 1 may be due to the mesophilic nature of the strains from which they were isolated.

Multiple sequence alignments (MSA) were generated using T-COFFEE Expresso, which also incorporates structural information (Armougom et al., 2006). Most cutinases (e.g., Cut190 and TfCut2) are designated as type I PETases, possessing one C-terminal disulfide bond, whereas type II PETases (e.g., *Is*PETase and PE-H) have an additional disulfide bond (Joo et al., 2018; Bollinger et al., 2020). Upon alignment with either the type I or type II PETases and visualization in ESPript, BgP was found to resemble the type I enzymes, with a single disulfide bond found near its terminal end (Supplementary Figures 2, 3). Given that BgP appears closely related to SM14est, based on their phylogeny, an alignment was also produced for these enzymes (Supplementary Figure 4). This indicated that 53% of their amino acids are identical, and a further 29% of their residues are biochemically similar.

**FIGURE 2:**
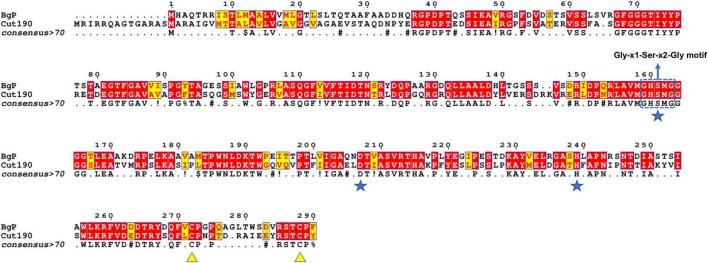
Amino acid sequence structural alignment of BgP and Cut190 generated with T-COFFEE Expresso and rendered using ESPript 3.0. Cut190 was indicated as a suitable structural homolog for BgP by the Phyre2 server. Amino acid residues shaded in red represent the ones strictly conserved between BgP and Cut190, while residues highlighted in yellow depict areas with an average level of homology. Catalytic triad residues are marked with a blue star, and disulfide bond cysteines are marked with a yellow triangle. The pentapeptide Gly-x1-Ser-x2-Gly serine hydrolase motif is outlined with a blue box.

Cut190 was selected for amino acid sequence comparison with BgP (Figure 2), based on the BLASTP sequence identities and Phyre2 predictions, where it was ranked as one of the top structural templates. From the resulting MSA, it was inferred that 54% of the amino acids in BgP were identical to Cut190, and that 83% of the BgP residues share similar biochemical properties to those of Cut190. The pentapeptide motif GHSMG is conserved in both sequences, together with the serine hydrolase catalytic triad (Ser-Asp-His). Both enzymes possess C-terminal cysteine residues, which can potentially join covalently to form a disulfide bond. The MSA had perfect agreement across all alignment methods used, with the output having an average consistency score of 100 (from 0 to 100).

Cut190 is a cutinase-like enzyme from *Saccharomonospora viridis* AHK190 that has been reported to hydrolyze PET (Kawai et al., 2014; Miyakawa et al., 2015). It is also a member of the lipase family and its thermostability and activity are enhanced by high concentrations of calcium ions, which are essential for the efficient enzymatic hydrolysis of amorphous PET (Miyakawa et al., 2015). Early mutagenesis studies of Cut190 found that an S226P/R228S substitution led to the highest activity and thermostability (Kawai et al., 2014). The resulting variant, named Cut190*, has been the target of multiple X-ray crystallography experiments and additional mutational analyses to elucidate the Ca^2+^-binding mechanism and further improve the variant for PET hydrolysis applications (Kawai et al., 2014; Miyakawa et al., 2015; Kawabata et al., 2017; Oda et al., 2018). Cut190*SS, a variant generated by combined mutation (Q138A/D250C-E296C/Q13H/N202H), increased the thermostability of Cut190* from 63 to 70°C, with a three-fold increase in PET film degradation. This suggests that there may be scope to undertake similar mutational modification of the BgP enzyme to generate variants with improved biochemical characteristics.

### Protein Homology Modeling for BgP and Comparative Analysis With Cut190

**FIGURE 3:**
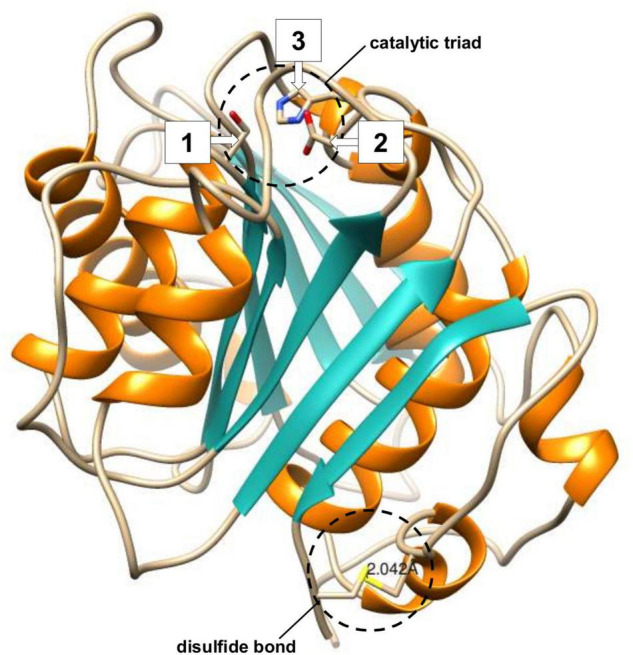
Three-dimensional structure of BgP based on the SeL (PDB code: 1JFR) template from *Streptomyces exfoliatus*, generated using UCSF-Chimera. Catalytic residues and disulfide bond cysteines are shown in stick form and circled. The calculated distance between the sulfur atoms of each cysteine is displayed in angstroms (Å). Secondary structures are shown as teal strands and orange helices, while coils are left uncolored.

Homology models that represent protein three-dimensional structure often offer insights into their conformation and functionality and enable the visualization of important features (Waterhouse et al., 2018). BgP was modeled using the SWISS-MODEL server, based on the top template, an enzyme named SeL, from *Streptomyces exfoliatus* (PDB code: 1JFR) (Figure 3). The signal peptide sequence of BgP was excluded in agreement with the SeL template, leaving 259 residues (32–290). The BgP model displayed a GMQE score of 0.87 (measured between 0 and 1, with higher numbers representing models with higher expected quality) and a QMEAN Z-score of −0.44 (with scores below −4.0 indicating low quality), indicating that the predicted model was reliable. Quality was also evaluated with tools from the SAVES suite, through which the BgP model was further validated. The ERRAT Overall Quality Factor was calculated to be 94.7% and a VERIFY averaged 3D-1D score (≥0.2) of 100%, exceeding the threshold values in both cases. The overall PROCHECK G-score was predicted to be −0.12 (negative value desired) and the ProSA Z-score was −8.15 (within native conformation range), with both indicating good model quality. PROCHECK Ramachandran plot calculations revealed 92.6% of amino acid residues in the most favored regions, with an additional 7% in allowed regions, and 0% in disallowed regions.

Structural analysis of the BgP model was completed in UCSF-Chimera allowing the key features to be located. The BgP enzyme possesses six full α-helices which surround a central β-sheet consisting of nine strands. This flexible core is a signature fold within the α/β hydrolase superfamily and is considered responsible for the multifunctionality of its member enzymes (Rauwerdink and Kazlauskas, 2015; Gricajeva et al., 2021). Active site residues Ser130, Asp176, and His208 are found in close proximity to each other, forming the catalytic triad that is typical of serine hydrolases. The active site lies at the apex of the β-sheet, which is consistent with polyesterases. All previously reported polyesterases display a Ser-Asp/Glu-His triad and follow the same catalytic mechanism where a nucleophilic serine initiates ester bond hydrolysis (Gricajeva et al., 2021). The Cys241 faces Cys257 at the C-terminal, bringing the residues into contact for disulfide linkage. The distance between these cysteine residues was measured to be 2.046Å, which corresponds to the typical length of a disulfide bond (Chaney and Steinrauf, 1974). Unlike cutinases and PETases, the lipase and esterase enzymes involved in polyester hydrolysis do not possess a disulfide bond. Both lipases and esterases have a lid-domain formed by at least two α-helices. Although the lid facilitates adsorption onto hydrophobic polyesters, it also covers the active site in these enzymes, which is buried relatively deeply within these enzymes. This lid structure is absent in most cutinases, which together with a more exposed active site near the enzyme surface, increases access to polyester substrates (Kawai et al., 2019). The *Streptomyces exfoliatus* template (1JFR) is an example of a cutinase-like lipase that lacks a lid domain (Khan et al., 2017), and this alpha-helical structure also appears to be absent from the BgP enzyme.

**FIGURE 4:**
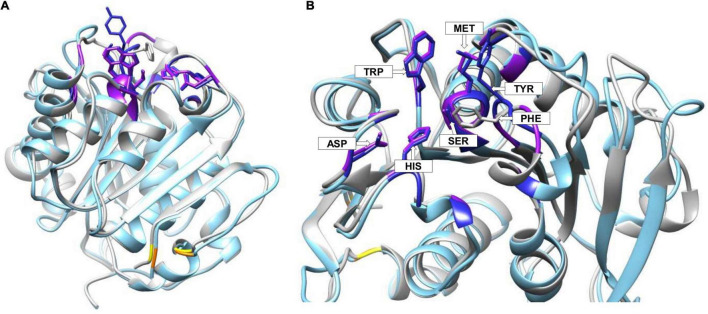
BgP structure (light blue) superimposed onto Cut190 (gray, PDB code: 4WFI) for comparative analysis. On the left view **(A)** the model is shown in full. On the right view **(B)**, a close-up of the active site pocket is shown. Potential ligand binding residues are displayed for BgP in dark blue, while any corresponding Cut190 residues, that are also conserved in BgP, are colored in purple. Key amino acid residues are shown in stick format, and in view **(B)** they are labeled using their 3-letter code. With the exception of tyrosine in BgP, which is replaced by phenylalanine in Cut190, these highlighted residues are identical in both enzymes. Disulfide bond cysteines are highlighted in yellow for Cut190 and in orange for BgP.

The Chimera MatchMaker tool was used for structural comparison of BgP with Cut190 (Figure 4). The Ser-Asp-His catalytic triad is conserved between the two enzymes, positioned as Ser176, Asp222, and His254 in Cut190, and as Ser130, Asp176, and His208 in BgP. Disulfide bond residues were positioned as follows; Cys241 and Cys257 in Cut190, and Cys287 and Cys302 in BgP. The polyesterase active site is found within a pocket called the substrate binding groove (SBG) (Gricajeva et al., 2021). There is also a conserved methionine adjacent to each nucleophilic serine, at position 177 in Cut190 and at 131 in BgP (Table 4). Met177 has been reported as an oxyanion hole-forming amino acid in Cut190 (Kawabata et al., 2017), while results from the LED database also point to Met131 as an oxyanion residue for BgP. The oxyanion hole stabilizes the reaction intermediate during polyester hydrolysis and is regarded as an important structural determinant of catalytic efficiency (Gricajeva et al., 2021). In Cut190, the oxyanion hole is formed by Met177 together with another residue, Phe106 (Kawabata et al., 2017). There is no corresponding phenylalanine residue implicated at this position in BgP, but instead another hydrophobic residue, Tyr62, is present in its place (Table 4). Tyrosine participates in oxyanion hole formation in certain α/β hydrolases and replaces phenylalanine in other PHEs such as TfCut2 and *Is*PETase, where it is believed to play a similar role in intermediate stabilization (Bauer et al., 2020; Tan et al., 2021). The importance of Phe106 for the activity of Cut190 is reflected in the fact that during mutational analysis of Cut190, the replacement of Phe106 with tyrosine led to decreased turnover of the model substrate poly(butylene succinate-co-adipate), or PBSA (Kawabata et al., 2017). Mutational and kinetic analyses will be required to investigate the potential role of Tyr62 in BgP during polyester hydrolysis, particularly if this residue has a similar impact on substrate turnover, as observed for Cut190.

**TABLE 4:** Comparative analysis of potential BgP binding residues, as predicted by 3DLigandSite, and the matching residue found at the same location in Cut190.

Predicted binding residue (BgP)	Corresponding residue (Cut190)	Predicted role (Cut190)^a^
Pro60	Pro104	Not fully investigated
Gly61	Gly105	Substrate interaction
Tyr62	Phe106	Substrate interaction
Thr63	Thr107	Not fully investigated
Ala64	Ala108	Not fully investigated
Ser68	Ser112	Only interacts in Ca^2+^-bound form
Ile69	Met113	Not investigated
Gln94	Gln138	Substrate interaction
Arg98	Arg142	Not fully investigated
His129	His175	Only interacts in Ca^2+^-bound form
Ser130	Ser176	Substrate interaction
Met131	Met177	Substrate interaction
Gly132	Gly178	Substrate interaction
Trp155	Trp201	Substrate interaction
Val178	Ile224	Substrate interaction
Ala179	Ala225	Not fully investigated
His208	His254	Only interacts in Ca^2+^-bound form
Leu209	Phe255	Only interacts in Ca^2+^-bound form
Asn212	Asn258	Only interacts in Ca^2+^-bound form

*^a^Based on mutational analysis of Cut190 (Kawabata et al., 2017).*

3DLigandSite, which employs structural results from the Phyre2 protein recognition server, was used to gain further insights into potential ligand-binding residues in BgP. Excluding the catalytic triad residues, 12 of the 16 binding residues predicted by 3DLigandSite were found to be conserved in Cut190, including hydrophobic Pro60, Gly61, Ala64, Met131, Gly132, Trp155, and Ala179. In polyesterases, hydrophobic amino acid residues found in the substrate-binding groove form crucial interactions with the substrate. Trp155 lies directly opposite the oxyanion residue Tyr62. The corresponding residue in Cut190, Trp201, was previously highlighted as having a role in substrate-binding, with low activity observed upon mutation of this residue; with the suggestion that binding is influenced by the indole ring in tryptophan and that the loss in activity following mutation was caused by weakened enzyme-substrate interactions (Kawabata et al., 2017). Along with Trp201, active site Ser176, and oxyanion Met177, results from the same study also indicated Gly105, Gln138, and Ile224 as likely Cut190 interacting residues using a partial PBSA structure called BABSBA. Gly105 and Gln138 are matched in BgP at positions 61 and 94, respectively. The Ile224 is replaced with a Val178 at the corresponding location in BgP. Although valine is smaller than isoleucine, both are hydrophobic amino acids, and share similar biochemical properties. In Cut190, mutation of Ile224 to Ala224, which is the smallest hydrophobic residue, was shown to increase activity despite a decrease in substrate affinity (Kawabata et al., 2017).

Cut190 has been shown to undergo a conformational change upon addition of Ca^2+^ ions, which bind to the enzyme and result in its activation (Oda et al., 2018). Three Ca^2+^-binding sites have been revealed by X-ray crystallography, involving the following amino acids; Ser76, Ala78, and Phe81 (site 1), Glu220, Asp250, and Glu296 (site 2), and Asp204 and Thr206 (site 3) (Oda et al., 2018). The serine and phenylalanine of site 1 are conserved in BgP (positions 31 and 37), together with the aspartate and threonine of site 3 (positions 158 and 160). Mutational analysis of Cut190 indicated that sites 1 and 3 are involved in activation, while sites 2 and 3 influence structural and thermal stability (Oda et al., 2018). It has also been reported that certain Cut190 residues only interact with the model substrate when the enzyme is in the Ca^2+^-bound state, namely Ser112, His175, Phe255, and Asn258 (Kawabata et al., 2017). In BgP, Ser68, His129, and Asn258 are conserved at the equivalent locations, with the exception of Phe255 which is replaced by Leu at BgP position 209. The presence of Ca^2+^ ions is an essential prerequisite for Cut190 in the context of PET polymer degradation, with this enzyme displaying active (Ca^2+^-bound) and inactive (Ca^2+^-free) states during PET hydrolysis (Kawai et al., 2014; Senga et al., 2021). The importance of metal ions (e.g., Ca^2+^ and Mg^2+^) for increased stability and degradation of PET has also been highlighted for other polyesterases, including TfCut2, and LCC (Sulaiman et al., 2014; Then et al., 2015). Given the similarities between BgP and Cut190, we expect it would be worth investigating the influence of metal ions on BgP functionality, with the potential to further increase activity by engineering of the ion-binding sites.

The glass transition temperature (*T*_g_) of PET is an important consideration for enzymatic hydrolysis, since its polymer chains display increased flexibility when reaction temperatures are set near or above *T*_g_ (Carr et al., 2020). For hydrolysis of amorphous PET under aqueous conditions, enzyme thermostability at 55°C, or preferably above 60°C, is recommended to facilitate efficient degradation, as exemplified by Cut190* (Kawai et al., 2014). During *in silico* protein analysis, BgP was classified as a stable protein based on the calculated instability index (32.44). The potential thermostability of BgP is indicated by its high aliphatic index (77.76), and its theoretical melting temperature (*T*_m_) of 69.4°C. However, further biochemical characterization of BgP will be required to determine its temperature profile and evaluate its stability under various reaction conditions. In an effort to determine potential targets for the engineering of improved BgP variants, the activity enhancing mutations in the aforementioned Cut190* and Cut190*SS may be valid targets. Based on conserved residues at these positions, the following mutations may be appropriate; S226P, R228S, Q138A, and N202H. However, a random mutagenesis approach may be more rewarding, given that there is no guarantee that mutations at these positions would generate similar results in BgP and considering that there might be other targets in the enzyme that prove more worthwhile.

### Cloning and Heterologous Expression of BgP

**FIGURE 5:**
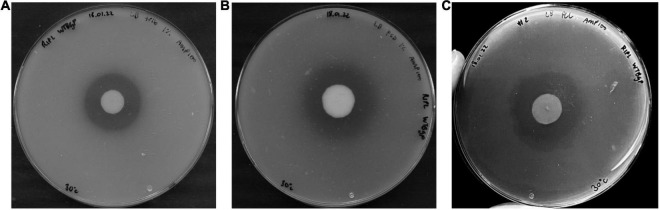
Heterologous expression by BL21 RIPL-(pET20b:BgP) clone plated on; **(A)** 1% tributyrin, **(B)** 1% PCD, and **(C)** 0.1% PCL, each prepared using LB agar and incubated for 6 days at 30°C.

To determine whether the *bgp* gene does encode polyesterase activity, it was amplified from B129SM11 genomic DNA using primers that were designed to incorporate the native *Brachybacterium ginsengisoli* signal peptide, and a C-terminal His_6_ tag. The gene was then cloned into the expression vector pET20b(+), generating the pET20b(+):BgP construct (Supplementary Figure 1). Following transformation into *E. coli* NEB^®^ 5-alpha, the construct was conjugated into the BL21 (DE3)-RIPL expression host. Polyesterase activity was confirmed using tributyrin, PCD, and PCL plate clearing assays (Figure 5), with zones observed on all three types of agar, indicating the hydrolysis of each substrate. The negative control, i.e., *E. coli* BL21 (DE3)-RIPL containing the pET-20b(+) plasmid without the insert did not display such activity toward any of the three substrates (Supplementary Figure 5).

In our previous study, SM14est was successfully exported by the *E. coli* heterologous host when its native *Streptomyces* sp. signal peptide was maintained in the expression construct (Almeida et al., 2019). We encountered some difficulties with BgP expression, although it is not certain whether this relates to the use of the native signal sequence in the expression construct. While zones of clearing were observed, they developed slowly. In this case, clearing may not indicate extracellular secretion, but rather leaky expression from the T7 promoter or perhaps enzyme release following natural cell lysis. The addition of IPTG was not shown to improve expression, or at least improvements were not detectable on the plate assays used, despite various attempts to optimize both induction time and IPTG concentration (Supplementary Figure 5), and this will therefore require further investigation. The efficient expression and secretion of polyesterases from the heterologous host will not only be important in assisting with protein purification and biochemical characterization of BgP, but it may also facilitate the use of polyesterase producers in bioremediation-type applications. To our knowledge, this is the first report of a cutinase-like polyesterase enzyme being identified in a deep-sea sponge-derived *Brachybacterium* isolate, which we hope will further expand our current knowledge of enzymes for the degradation of synthetic polyesters.

## Concluding Remarks

Polyester-degrading enzymes, which have to date been reported as either lipase, carboxylesterase, or cutinase family members, have become the subject of extensive research to achieve enzymatic hydrolysis of synthetic polyester plastics such as PET. Given the relatively recent development of synthetic plastic products and the exponential increase in plastic production and pollution that followed, the study of PET hydrolase enzymes has quickly gained traction. A number of PET-hydrolyzing polyesterases have to date been identified and characterized, which together with efforts to elucidate the mechanisms behind functionality and the engineering of improved variants, has advanced the field to the extent that the practical implementation of biological PET degradation systems is now being examined. However, there are many factors preventing the complete hydrolysis of PET by polyesterases, particularly due to the individual properties of different PET products and in particular due to the fact that most of our knowledge to date is limited to the enzyme’s interactions with amorphous PET films and with model substrates. Furthermore, much of the research in this area is focused on PET biorecycling applications, with less known about polyesterases in the context of environmental degradation and potential for enzymatic remediation.

While most PET polyesterases that have been reported to date have primarily been from Actinobacteria, we report here on a novel polyesterase from a member of the genus *Brachybacterium* which was isolated from the deep-sea sponge *Pheronema* sp. at a depth of 2,129 m. Activity testing and subsequent genome mining of *B. ginsengisoli* B129SM11 resulted in the identification of BgP, a cutinase-like polyesterase that successfully hydrolyzed tributyrin, polycaprolactone, and polycaprolactone diol substrates following heterologous expression in *E. coli*. Important functional and mechanistic insights were gained through comparisons with known PET hydrolases and by protein modeling of BgP. This work lays the foundation for future biochemical characterization and kinetic analysis, as well as mutational studies to determine structure-function relationships to potentially improve the enzyme for polyester hydrolysis and other biocatalytic applications. Furthermore, it would be interesting to explore the possible role of BgP in *B. ginsengisoli* in the context of its native deep-sea ecosystem, and specifically its function within the sponge microbiome. That said, we should consider that hydrolases are highly promiscuous, and enzymes such as BgP may not have specifically evolved to degrade PET, and its involvement in PET degradation would require additional enzymes and transporters as part of a specific PET catabolic pathway. Nonetheless, further exploration of the role of BgP will broaden our understanding of polyesterases in marine environments and potentially facilitate the development of bioremediation-based applications in these ecosystems.

## Data Availability Statement

The datasets presented in this study can be found in online repositories. The names of the repository/repositories and accession number(s) can be found in the article/Supplementary Material.

## Author Contributions

CC, BO, SJ, and AD conceived and designed the experiments. CC performed the experimental work. CC and BO analyzed the data. SJ and AD contributed reagents, materials, and analysis tools. CC, BO, and AD wrote the manuscript. AD and DC supervised the study. All authors read and approved the final manuscript.

## Conflict of Interest

The authors declare that the research was conducted in the absence of any commercial or financial relationships that could be construed as a potential conflict of interest.

## Publisher’s Note

All claims expressed in this article are solely those of the authors and do not necessarily represent those of their affiliated organizations, or those of the publisher, the editors and the reviewers. Any product that may be evaluated in this article, or claim that may be made by its manufacturer, is not guaranteed or endorsed by the publisher.

## Footnotes

1
http://www.bioinformatics.babraham.ac.uk/projects/fastqc

2
http://www.led.uni-stuttgart.de/

3
https://www.ebi.ac.uk/interpro/search/sequence/

4
http://pfam.xfam.org/

5
https://supfam.mrc-lmb.cam.ac.uk/SUPERFAMILY/

6
https://saves.mbi.ucla.edu

7
https://www.snapgene.com/

8
https://www.genscript.com/tools/restriction-enzyme-map-analysis

9
https://molbiotools.com/restrictionanalyzer.php

10
http://www.oligoevaluator.com/

11
https://www.thermofisher.com/ie/en/home/brands/thermo-scientific/molecular-biology/molecular-biology-learning-center/molecular-biology-resource-library/thermo-scientific-web-tools/multiple-primer-analyzer.html

12
https://tmcalculator.neb.com/
