# Supplementary material for: Identification of BgP, a Cutinase-Like Polyesterase From a Deep-Sea Sponge-Derived Actinobacterium
Source: Front Microbiol. 2022 Apr 12;13:888343. doi: 10.3389/fmicb.2022.888343 (PMC9039725; doi:10.3389/fmicb.2022.888343)

Supplementary Material

1 Supplementary Figures and Tables

Table S1. Overall genome features of *Brachybacterium ginsengisoli* B129SM11.

Genome property	Value
Size (bp)	3,987,864
Number of contigs	22
Largest contig (bp)	898,629
N50	651,468
L50	3
GC content (%)	71.55
Completeness (%)	100
Contamination (%)	0.58
Number of coding sequences	3,505
Number of hypothetical proteins	1,592
Number of rRNA genes	3
Number of tRNA genes	58
Number of tmRNA genes	1
Number of miscellaneous RNA genes	13

Figure S1. Vector map of the pET20b(+):BgP plasmid construct for expression of BgP in *E. coli* heterologous hosts. The insert and key features of the vector are graphically represented and labelled accordingly.

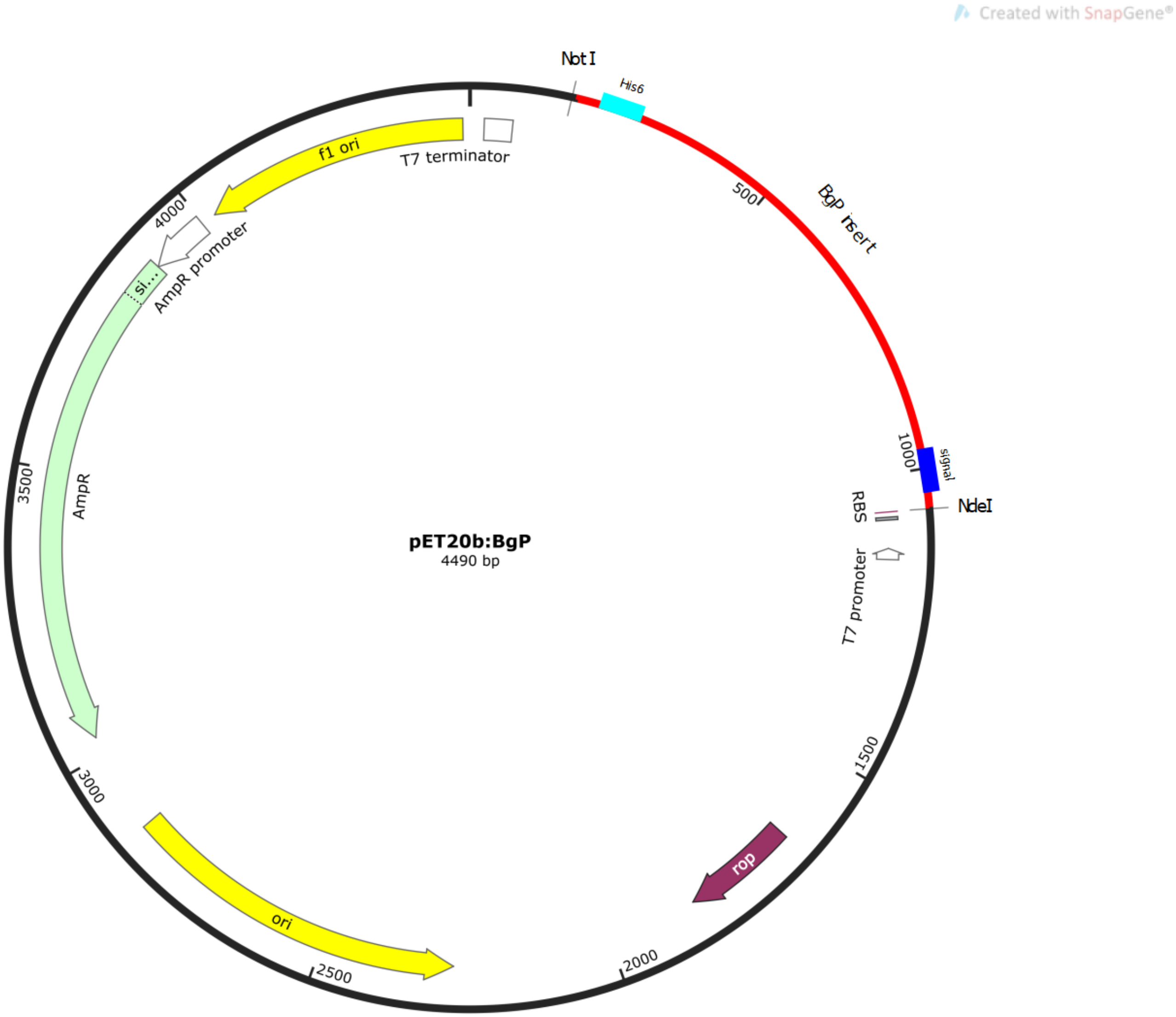

Figure S2. Amino acid sequence structural alignment of BgP and some “type I” PETases, generated with T-COFFEE Expresso and rendered using ESPript 3.0. Amino acid residues shaded in red depict strictly conserved residues, while residues highlighted in yellow represent areas with an average level of homology. Disulfide bond cysteines are marked with a yellow triangle.

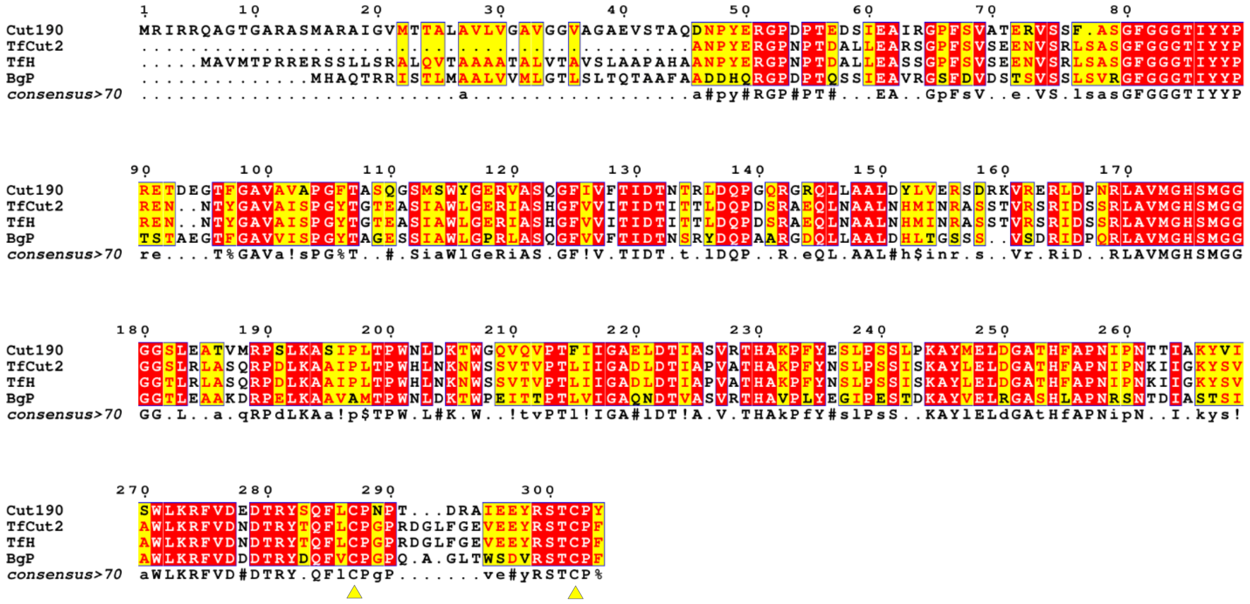

Figure S3. Amino acid sequence structural alignment of BgP and some “type II” PETases, generated with T-COFFEE Expresso and rendered using ESPript 3.0. Amino acid residues shaded in red depict strictly conserved residues, while residues highlighted in yellow represent areas with an average level of homology. The cysteine residues forming disulfide bond 1, which is common to both PETase types, are marked with a yellow triangle, while cysteines forming disulfide bond 2, which is specific to type II PETases, are marked with an orange triangle.

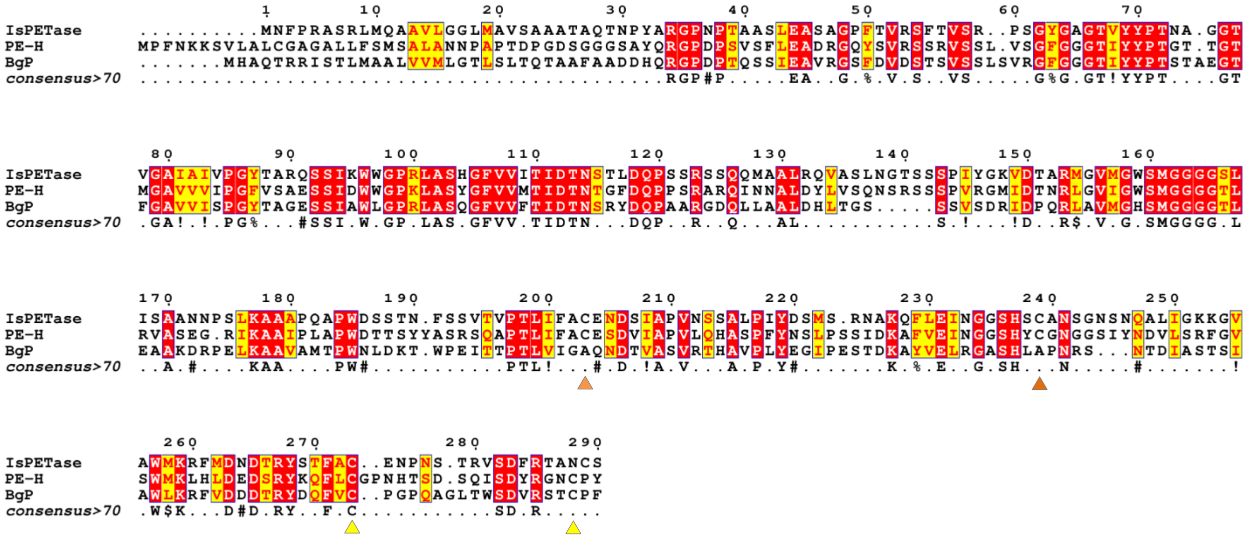

Figure S4. Amino acid sequence structural alignment of BgP, and SM14est from marine sponge-derived *Streptomyces* sp. SM14, generated with T-COFFEE Expresso and rendered using ESPript 3.0. Amino acid residues shaded in red depict strictly conserved residues, while residues highlighted in yellow represent areas with an average level of homology.

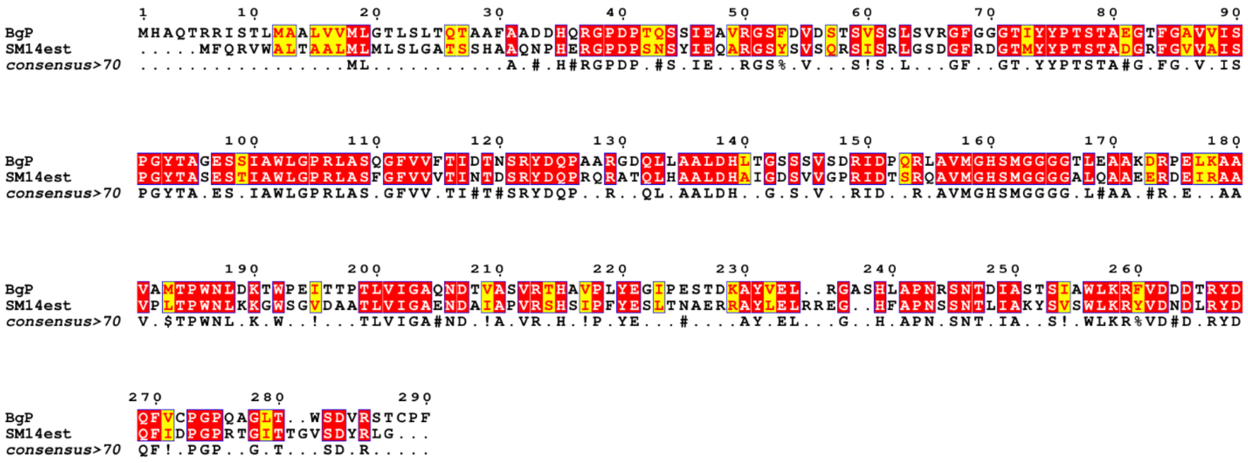

Figure S5. BL21-Codon Plus (DE3)-RIPL *E. coli* negative control (labelled NC), growing on; (A) tributyrin, (B) PCD, and (C) PCL. In the case of (A) and (B), the negative control is plated alongside the BgP production strain, which was being investigated under three different conditions, labelled 1, 2, and 3 (where 1 = no IPTG added, 2 = 0.5 mM IPTG added, 3 = 1 mM IPTG added).

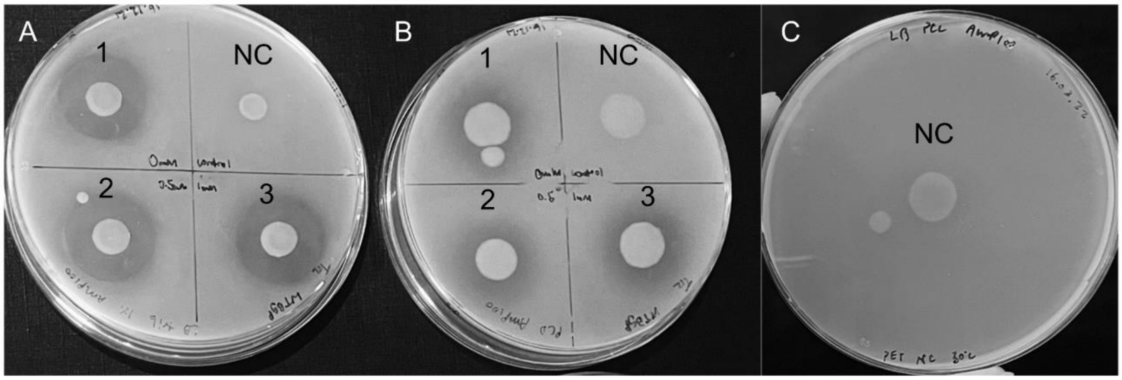

Supplement: Supplementary file 1 [file Data_Sheet_1.pdf]
